# Evolution of *GOLDEN2*-*LIKE* gene function in C_3_ and C_4_ plants

**DOI:** 10.1007/s00425-012-1754-3

**Published:** 2012-09-12

**Authors:** Peng Wang, Jim Fouracre, Steven Kelly, Shanta Karki, Udo Gowik, Sylvain Aubry, Michael K. Shaw, Peter Westhoff, Inez H. Slamet-Loedin, W. Paul Quick, Julian M. Hibberd, Jane A. Langdale

**Affiliations:** 1 Department of Plant Sciences, University of Oxford, South Parks Rd., Oxford, OX1-3RB UK; 2 IRRI, Los Banos, Philippines; 3 Institut für Entwicklungs- und Molekularbiologie der Pflanzen, Heinrich-Heine-Universität, Universitätsstr. 1, 40225 Düsseldorf, Germany; 4 Department of Plant Sciences, University of Cambridge, Downing St., Cambridge, CB2-3EA UK; 5 Sir William Dunn School of Pathology, University of Oxford, South Parks Rd., Oxford, OX1-3RE UK

**Keywords:** Bundle sheath, Chloroplast, Cleome, Mesophyll, Rice, Sorghum

## Abstract

A pair of GOLDEN2-LIKE transcription factors is required for normal chloroplast development in land plant species that encompass the range from bryophytes to angiosperms. In the C_4_ plant maize, compartmentalized function of the two *GLK* genes in bundle sheath and mesophyll cells regulates dimorphic chloroplast differentiation, whereas in the C_3_ plants *Physcomitrella patens* and *Arabidopsis thaliana* the genes act redundantly in all photosynthetic cells. To assess whether the cell-specific function of *GLK* genes is unique to maize, we analyzed gene expression patterns in the C_4_ monocot *Sorghum bicolor* and C_4_ eudicot *Cleome gynandra*. Compartmentalized expression was observed in *S. bicolor*, consistent with the development of dimorphic chloroplasts in this species, but not in *C. gynandra* where bundle sheath and mesophyll chloroplasts are morphologically similar. The generation of single and double mutants demonstrated that *GLK* genes function redundantly in rice, as in other C_3_ plants, despite the fact that *GLK* gene duplication in monocots preceded the speciation of rice, maize and sorghum. Together with phylogenetic analyses of *GLK* gene sequences, these data have allowed speculation on the evolutionary trajectory of GLK function. Based on current evidence, most species that retain single *GLK* genes belong to orders that contain only C_3_ species. We therefore propose that the ancestral state is a single *GLK* gene, and hypothesize that *GLK* gene duplication enabled sub-functionalization, which in turn enabled cell-specific function in C_4_ plants with dimorphic chloroplasts. In this scenario, *GLK* gene duplication preconditioned the evolution of C_4_ physiology that is associated with chloroplast dimorphism.

**Electronic supplementary material:**

The online version of this article (doi:10.1007/s00425-012-1754-3) contains supplementary material, which is available to authorized users.

## Introduction

Chloroplast differentiation in flowering plants is influenced by both environmental and developmental cues. From a developmental perspective, a major difference is seen between chloroplast differentiation in C_3_ and C_4_ plants. In C_3_ plants, a single chloroplast type develops in all photosynthetic cells, whereas in many C_4_ plants, dimorphic chloroplasts are formed in distinct bundle sheath (BS) and mesophyll (M) cells (reviewed in Langdale 2011). C_3_ chloroplasts accumulate Ribulose Bisphosphate Carboxylase/Oxygenase (RuBisCO), fix CO_2_ in the Calvin-Benson cycle and form stacked thylakoids. Consistent with the fact that C_4_ photosynthesis evolved from C_3_ during land plant evolution (reviewed in Sage et al. 2011), chloroplasts in C_4_ plants differentiate a C_3_ state by default. However, in the presence of light, and in cells within a two-cell radius of a vein, distinct C_4_ BS and M chloroplasts develop (Langdale et al. 1988b). In the BS cells that are immediately adjacent to the veins, chloroplasts accumulate RuBisCO, the Calvin-Benson cycle operates and thylakoid membranes are often (but not always) unstacked. In contrast, M cell chloroplasts develop stacked thylakoids and RuBisCO is absent. Distinct regulatory mechanisms must therefore operate in BS and M cells of C_4_ plants to control chloroplast development.

Very few transcriptional regulators of chloroplast development have been reported in either C_3_ or C_4_ plants. Of those identified, GOLDEN2-like (GLK) transcription factors were first characterized in the C_4_ plant maize (Hall et al. 1998). *GLK* genes are members of the GARP superfamily (Riechmann et al. 2000) and in maize each member of a paralogous *GLK* gene pair (*ZmG2* and *ZmGlk1*) functions in a BS or M cell-type specific manner to regulate the proplastid to chloroplast transition (Langdale and Kidner 1994; Hall et al. 1998; Rossini et al. 2001). The *ZmG2* gene is expressed in BS cells whereas Z*mGlk1* is expressed in M cells. The extent to which compartmentalization of *GLK* gene function in maize is representative of a more general C_4_ regulatory mechanism has not yet been investigated.

*GLK* gene pairs have also been identified in the C_3_ moss *Physcomitrella patens* (Yasumura et al. 2005; Bravo-Garcia et al. 2009), the eudicot *Arabidopsis thaliana* (Fitter et al. 2002; Tamai et al. 2002; Waters et al. 2009) and the monocot *Oryza sativa* (Rossini et al. 2001; Nakamura et al. 2009). In all three cases, both members of the gene pair are expressed in all photosynthetic cells. In *P. patens* and *Arabidopsis*, this expression pattern reflects redundant gene function because chloroplast differentiation is not perturbed unless both gene copies are mutated. Unfortunately, the maize, moss and *Arabidopsis* genes are not orthologous and thus evolutionary trajectories of gene function cannot be inferred from these mutant phenotypes.

In rice, *OsGLK1* is an ortholog of *ZmGlk1* and *OsGLK2* is an ortholog of *ZmG2* (Rossini et al. 2001). As such, *GLK* gene duplication in this lineage preceded the speciation of rice and maize. It is thus possible that *GLK* gene function was sub-functionalized prior to the divergence of the two species. If this were the case, mutations in individual *GLK* genes would perturb aspects of chloroplast development in rice. An alternative hypothesis is that *GLK* gene duplication preconditioned compartmentalized C_4_ function in maize (and perhaps other C_4_ species) but that in rice the duplicated genes act redundantly. In this case, chloroplast development in rice would only be perturbed in double mutants, as in *Arabidopsis* and moss.

To provide more insight into the evolutionary trajectory of *GLK* gene function in land plants, we have examined the phylogeny of *GLK* genes in the context of the current plant genome sequence database, have investigated the expression profile of *GLK* genes in two more C_4_ species, and have determined the phenotypic effect of perturbed *GLK* gene function in rice. Our results suggest that *GLK* gene duplications were primarily associated with the numerous genome-wide duplications that occurred within the angiosperms. We propose that the retention of multiple *GLK* copies in the genomes of both C_3_ and C_4_ species reflects sub-functionalization.

## Materials and methods

### Plant material and growth conditions

*Cleome gynandra* L. (Millenium Seedbank, Kew) plants were grown for 10 days in soil under long-day conditions with fluence rates of 150 µmol photon m^−2^ s^−1^ and a temperature of 23 °C.

*Sorghum bicolor* L. Moench inbred line BTx623 (USDA-ARS-SPA, Lubbock, TX, USA) was used as the genetic background for northern blot analyses. Sorghum plants were grown in soil in a greenhouse, with the natural diurnal light period in Oxford (UK), and were supplemented with 500 µmol photon m^−2^ s^−1^ when necessary, and up to 14 h in winter. The average daytime temperature was 28 °C and the average night temperature was 20 °C. *Sorghum bicolor* L. hybrid line Tx430 (Pioneer Hi-Bred, Plainview, TX, USA) was used as the genetic background for Illumina sequencing. Plants were grown in soil in a greenhouse, with the natural diurnal light period in Duesseldorf (Germany) and were supplemented with 300 µmol photon m^−2^ s^−1^ when necessary, and up to 14 h in winter. Average daytime temperature was 25 °C and average night temperature was 19 °C.

*Oryza sativa* var. japonica cv. Dongjin was used as the genetic background for all rice experiments. Rice plants were grown as described for the BTx623 sorghum line. *Osglk1* and *Osglk2* single mutants were grown and crossed in the glasshouse at the International Rice Research Institute (IRRI, Los Banos, Philippines). T_1_ seeds of the *Osglk1*-*2* single mutant and T_3_ homozygous seeds of the *Osglk2*-*2* mutant were incubated at 45 °C for 5 days to break seed dormancy, germinated on MS medium in petri dishes at 30 °C for 7 days, and then transplanted to pots containing soil. Plants were grown with a day/night temperature of 30/22 ± 3 °C and 65–85 % relative humidity. *Osglk1*-*2* single mutants were PCR screened for the RNAi transgene and only PCR-positive plants were transplanted to pots. One-third of these plants should be homozygous for the transgene and two-thirds should be heterozygous.

### Phylogenetic inference

To identify *GLK* genes, BLASTP was used to search all of the annotated land plant proteomes on Phytozome v8.0 (http://www.phytozome.net) plus the potato genome sequence (http://potatogenomics.plantbiology.msu.edu/), using the *Zm*GLK1 amino acid sequence as a query. Results for searches against each proteome were filtered manually to identify *GLK* genes (distinguished from other GARP family genes by an AREAEAA motif (consensus motif) at the C terminal of the DNA-binding domain). To ensure that all putative *GLK* genes were identified the amino acid sequences encoded by 5 *GLK* genes representing a wide range of angiosperm lineages (*AtGLK1*, *GmGLKD*, *VvGLK*, *ZmGlk1*, *OsGLK2*) were aligned using MAFFT (Katoh et al. 2005). This alignment was converted to a hidden Markov model and used to search Phytozome v8.0 plant and algal proteomes with an iterative HMMer search algorithm described previously (Eddy 1998; Kelly et al. 2011).

Phylogenetic trees of the identified *GLK* genes were inferred using both Bayesian and maximum likelihood methods. Protein sequences were aligned using MergeAlign (Collingridge and Kelly 2012). A 100 bootstrap maximum likelihood tree was inferred using RAxML (Stamatakis 2006) employing the LG model of sequence evolution (Le and Gascuel 2008) and CAT rate heterogeneity. A 50 % majority-rule consensus tree was calculated from the 100 bootstrap replicates using the python module dendropy (Sukumaran and Holder 2010). Bayesian phylogenetic trees were inferred using mrbayes v3.1.2 (Huelsenbeck and Ronquist 2001) with gamma-distributed substitution rate variation approximated by four discrete categories and shape parameter estimated from the data. The “covarion” model (Galtier 2001) was implemented and four chains were employed, each with a temperature of 0.2. Tree inference was made from a random start tree and allowed to run for 2,500,000 generations. The time taken to reach stationary phase was approximately 700,000 generations and thus the final 1,800,000 trees sampled every 200 generations were used to infer posterior probabilities on topology.

### Identification of *Osglk2* insertional mutants

*Osglk2* T-DNA insertion lines (PFG-3A-13668.L) were ordered from RiceGE: Rice Functional Genomic Express Database http://signal.salk.edu/cgi-bin/RiceGE (An et al. 2003). 15 lines of T_2_ seeds were received (PFG-3A-13668-01 to PFG-3A-13668-15). DNA was extracted from five seedlings of each line, and PCR was performed using forward (5′-CAATTATGCGGTAGCAGCTG-3′) and reverse (5′-TCTCTGTCCAATAAAATCGAACTTC-3′) primers flanking the insertion, and a T-DNA right border primer (5′-AACGCTGATCAATTCCACAG-3′). The forward and reverse primers were used as a pair to generate a 1,072-bp fragment of the wild-type allele. The forward primer and T-DNA right border primer were used as a pair to generate a shorter fragment of the insertion allele. PCR conditions were 35 cycles of: 95 °C for 30 s, 53 °C for 30 s, 72 °C for 1.5 min. Lines containing the insertion allele were carried through to DNA gel blot analysis.

### Generation of *Osglk1* RNAi mutant lines

*Osglk1* single mutant lines were generated by RNAi knock down of the *OsGLK1* gene (Os06g24070) in *O. sativa* Dongjin. A 305-bp sequence of the *OsGLK1* GCT-box (fragment 2 in Fig. 4a) was used as the target sequence. The sequence was first inserted downstream of the potato GA20 oxidase intron in the pUC-RNAi vector (Fang et al. 2008), as a BamHI/XbaI fragment in the sense orientation. The same sequence was then inserted in the antisense orientation into the BglII/SpeI sites of the pUC-RNAi construct that contained the sense fragment. To create the binary construct, the fragment comprising sense and antisense sequences of *OsGLK1,* separated by the potato GA20 oxidase intron, was excised from pUC-RNAi and inserted into the *Pst1* site of pXQAct (Fang et al. 2008) between the rice *actin1* promoter and *Ocs* terminator. *Agrobacterium*-mediated transformation into wild-type Dongjin callus was performed as described (Nishimura et al. 2006). After selection with G418 and PCR validation, seven regenerated plants were obtained that contained the RNAi construct.

### Generation of *Osglk1,glk2* double-mutant lines

To generate a double mutant, a 395-bp sequence between the *OsGLK1* gene DNA-binding domain and GCT-box (fragment 1 in Fig. 4a) was used to create an RNAi construct as shown earlier. This construct was transformed into *Osglk2*-*2* mutant callus. After selection with G418 and PCR validation, 20 regenerated plants were obtained that contained the RNAi construct. Unfortunately, none of the regenerated double mutants produced viable seed. An F_2_ population that segregated double mutants was therefore generated by crossing a homozygous *Osglk2*-*2* single mutant line with a hemizygous *Osglk1*-*2* knockdown line. The resultant F_1_ progeny were selfed to generate a segregating F_2_ population.

### Isolation of BS and M cells

For northern blot analysis, BS and M cells were separated from fully expanded 3rd leaves of *S. bicolor* inbred line BTx623. M cells were separated enzymatically from leaf tissue essentially as described by Sheen and Bogorad (1985), but with vanadyl ribonucleoside complex omitted from the protoplast washing buffer. Bundle sheath strands were isolated mechanically using a household blender. Leaves were blended and filtered through 60 µM mesh using buffers described by Westhoff et al. (1991). Cell preparations were checked microscopically for purity and immediately frozen in liquid nitrogen before storage at −80 °C. For Illumina sequencing, M and BS cells were separated enzymatically as described previously (Wyrich et al. 1998).

*C. gynandra* BS and M cells were isolated by laser capture microdissection (LCM). Mature leaf tissue was harvested 4 h after dawn and immediately infiltrated with ethanol: acetic acid (3:1, v/v). The tissue was processed through a dehydration series of ethanol and Histoclear and then replaced by Paraplast Xtra. Leaf sections were floated in ethanol on MembraneSlide 1.0 PEN (Zeiss). LCM was performed using Arcturus XT (Life Technologies) and M and BS cells were captured using HS adhesive caps (Life Technologies) following the manufacturer’s instructions.

### DNA and RNA analysis

Genomic DNA was isolated using a modified CTAB method (Murray and Thompson 1980). Total leaf RNA was isolated by guanidinium thiocyanate–phenol–chloroform extraction as described by Waters et al. (2008). RNA was extracted from separated sorghum BS and M cells as described by Sheen and Bogorad (1985) (for northern blot analysis) or by Wyrich et al. (1998) (for Illumina sequencing).

Total RNA from BS or M cells of *C. gynandra* harvested by LCM was extracted from three independent replicates using a Picopure RNA isolation kit (Life Technologies) and DNAse treatment. RNA integrity was assessed on a Bioanalyzer 2100 RNA picochip (Agilent). At least 5 ng of RNA for each sample was subsequently amplified through two rounds of amplification using the RiboAmp HS plus RNA amplification kit (Life Technologies).

For Illumina sequencing, RNA from five cell preparations of 10-day-old sorghum seedlings was pooled and the mRNA content was purified using the Oligotex mRNA Midi Kit (Qiagen). cDNA was produced using the SMARTer PCR cDNA Synthesis Kit (Clontech) and sent to GATC Biotech AG (Konstanz, Germany) for 40 bp Illumina sequencing using a standard library preparation protocol. Following standard GATC quality filtering, raw reads were mapped to sorghum Sbi1_4 gene models (http://genome.jgi-psf.org/Sorbi1/Sorbi1.info.html) using Bowtie 0.12.8 (Langmead et al. 2009) in the –v alignment mode with up to 3 mismatches and the –best option activated. Differentially expressed genes were calculated using a significance test (Audic and Claverie 1997) followed by a Bonferroni correction.

For real-time PCR, first-strand cDNA was synthesized from 5 ng amplified RNA using Superscript II (Invitrogen). Real-Time PCR was performed using SYBRgreen Jumpstart (Sigma) in a rotor-gene-Q system (Qiagen). Relative transcript levels were calculated based on *Actin 7* levels. Primer sequences were as follows—*CgGLK1:* 5′-TCCGACTTGTGCACCGTATGATGT-3′ and 5′-ACCGAATGCCAAATGGAACGACAC-3′; *CgGLK2:* 5′-AAAGTTACGGGAGACGGTGGGAAA-3′ and 5′-CACGAATTTCCGGTGCAATTCCGA-3′; *CgACT7:* 5′-TCCGACCCGATGTGATGTTATGGT-3′ and 5′-CAATCACTTTCCGGCTGCAACCAA-3′.

DNA and RNA gel blots were prepared and hybridized in 0.45 M NaCl at 65 °C as described previously (Langdale et al. 1988a), using gene-specific probes as follows: *SbGLK1* (transcript bases 1558–1864), *SbGLK2* (transcript bases 2029–2346), *ZmPEPC* (pTN1, Langdale et al. 1988a), *ZmRbcS* (pJL10, Langdale et al. 1988a), *OsGLK1* (transcript bases 1543–1856), *OsGLK2* (transcript bases 2044–2325), NPTII, GUS (290 bp from the 5′ end of the cDNA amplified using primers 5′-ATGTTACGTCCTGTAG-3′ and 5′-ACTTTGCCGTAATGAGTGACC-3′). Blots were visualized and quantified using a Molecular FX phosphorimager (Bio-Rad, http://www.bio-rad.com/).

### Light and transmission electron microscopy

For light microscopy, thick sections were prepared according to Yamada et al. (2009). One-month-old leaf blades were vacuum infiltrated for 10 min with fixation buffer [50 mM PIPES–NaOH, pH 6.9, 4 mM MgSO_4_, 10 mM EGTA, 0.1 % (w/v) Triton X-100, 200 µM phenylmethylsulfonyl fluoride, 5 % (v/v) formaldehyde and 1 % (v/v) glutaraldehyde] and then incubated at 4 °C overnight. The fixed segments were then embedded in 5 % (w/v) agar and sectioned at 70–80 µm with a Vibratome Series 1000 Sectioning System. Alternatively, leaf samples were fixed overnight in FAA (4 % formaldehyde, 5 % acetic acid, 50 % ethanol) and embedded in Paraplast Plus. Thin sections (8 µm) were cut using a rotary microtome and stained with Safranin/Fast Green as described previously (Langdale 1994). Sections were viewed and photographed with a Leica DMRB microscope.

For transmission electron microscopy, leaf samples were fixed in the dark by immersion in ice-cold fixative (4 % paraformaldehyde, 3 % glutaraldehyde in 0.05 M potassium phosphate buffer, pH 7) followed by vacuum infiltration. Subsequent steps were performed as described previously (Waters et al. 2008). Samples were stained sequentially with 2 % w/v OsO_4_ and 0.5 % w/v uranyl acetate and embedded in TAAB 812 resin (TAAB Laboratory Equipment, http://www.taab.co.uk). 0.1 µm sections were stained with 0.2 % w/v lead citrate, rinsed in deionized water, and then examined using a Zeiss (LEO) Omega 912 electron microscope. Digital images were captured using the SIS package (Soft Imaging Software GmbH, http://www.soft-imaging.net).

### Chlorophyll assays

Chlorophyll was extracted from 2-month-old rice plants with replicates from four different plants assayed per line. Leaf tissues of the same fresh weight (200 mg) were ground in liquid nitrogen and resuspended in 80 % acetone. After incubation overnight in the dark at 4 °C, cell debris was pelleted by centrifugation for 1 min at 15,000*g* and the absorbance of the supernatant was measured at 663 and 645 nm on a Unicam UV4 UV/Vis Spectrometer. Total chlorophyll was calculated as (8.02 × A663 + 20.29 × A645) × *V*/1,000 × *W*, where *V* = volume of the extract (ml); *W* = weight of fresh leaves (g) (Arnon 1949).

## Results

### *GLK* gene phylogeny

**Fig. 1:**
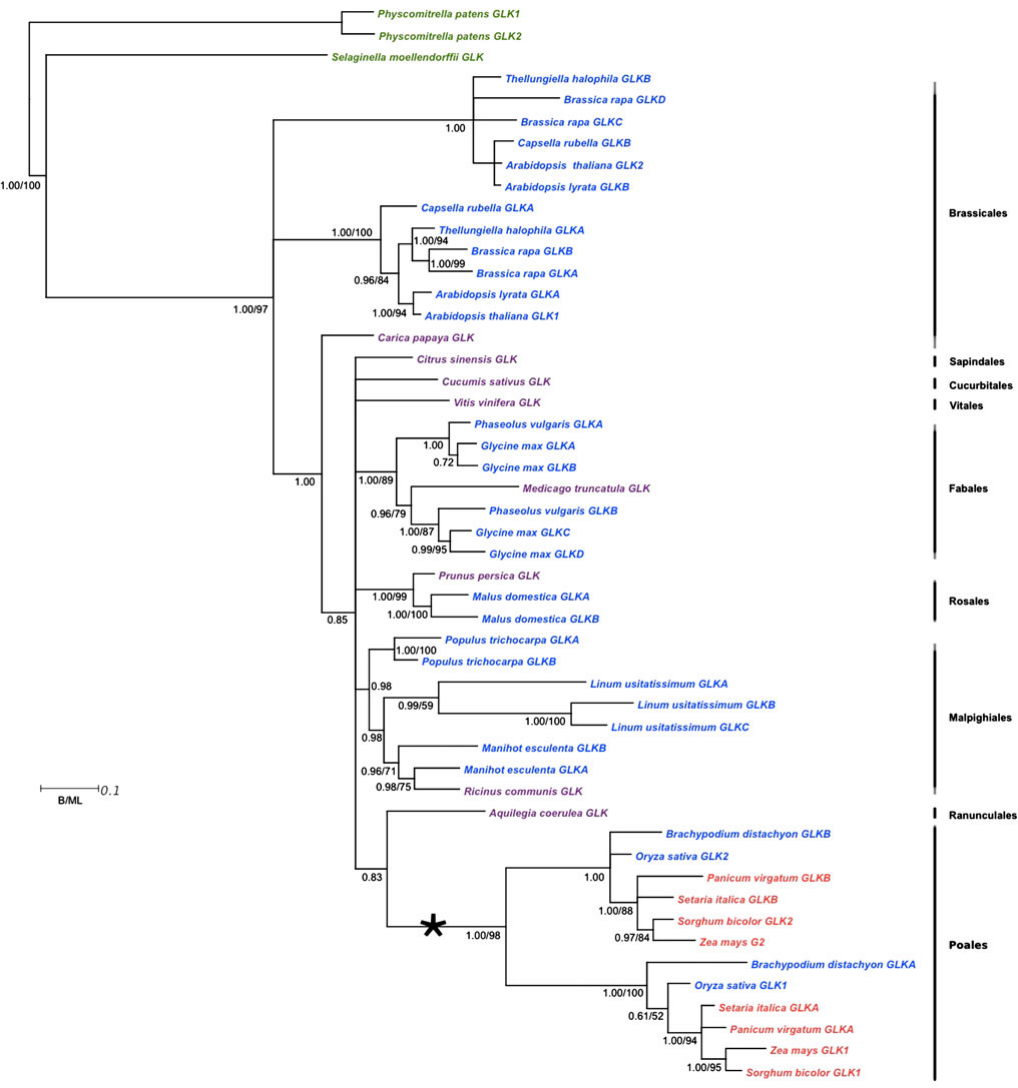
*GLK* gene phylogeny. Bayesian phylognetic tree of GLK genes. Posterior probabilities (B) and bootstrap support values (ML) where appropriate are shown at branch nodes. Sequences highlighted in *green* are non-angiosperm *GLK* genes, in *purple* are C_3_ species with a single *GLK* gene, in *blue* are C_3_ species with *GLK* duplicates and in *red* are C_4_ species with *GLK* duplicates. Characterized *GLK* genes are annotated with *numbers* (i.e. *GLK1*, *GLK2*), those that have not been previously described are annotated with *letters* (i.e. *GLKA*-*GLKD*). *GLK* sequences correspond to the gene accessions shown in Supplemental Table S1. *Asterisk* indicates gene duplication in the Poales

To determine the *GLK* gene phylogeny, annotated plant genomes were searched using *Zm*GLK1 as a query sequence. *GLK* genes are distinguished from other members of the GARP family by the presence of a C terminal GCT-box and by an AREAEAA motif (consensus sequence) at the C terminal of the DNA-binding domain (Fitter et al. 2002). 57 *GLK* genes were identified (Supplemental Table S1). To confirm that *GLK* genes were not overlooked during manual searching, an alignment of a subset of *GLK* genes was used as a template for an iterative HMMer search of the 31 genomes used (Kelly et al. 2011). Phylogenetic analyses showed that 56 of the 57 identified *GLK* genes form a monophyletic clade that is a sister group to the pseudo-response regulator (PRR) group of GARP family genes (data not shown). The single *Selaginella moellendorffii GLK* gene clustered with the PRR genes due to the additional presence of a pseudo-response regulator receiver domain in the *S. moellendorffii GLK* gene sequence. Crucially, no new *GLK* genes were identified. Phylogenetic trees of the 57 *GLK* gene sequences were generated using Bayesian and maximum likelihood methods. Preliminary phylogenetic analyses suggested long-branch attraction in the *Eucalyptus, Mimulus,* and potato sequences and thus they were removed from subsequent analyses. The tree based on the remaining 50 GLK genes (Fig. 1) demonstrates two key points. First, all four C_4_ species in the dataset have two *GLK* genes (colored red). Second, some C_3_ species have a single *GLK* gene (colored purple), whereas others have two or more *GLK* genes (colored blue). These data are consistent with the suggestion that the last common ancestor of flowering plants had a single *GLK* gene and that gene duplication occurred in specific lineages.

### *GLK* gene expression in C_4_ plants

**Fig. 2:**
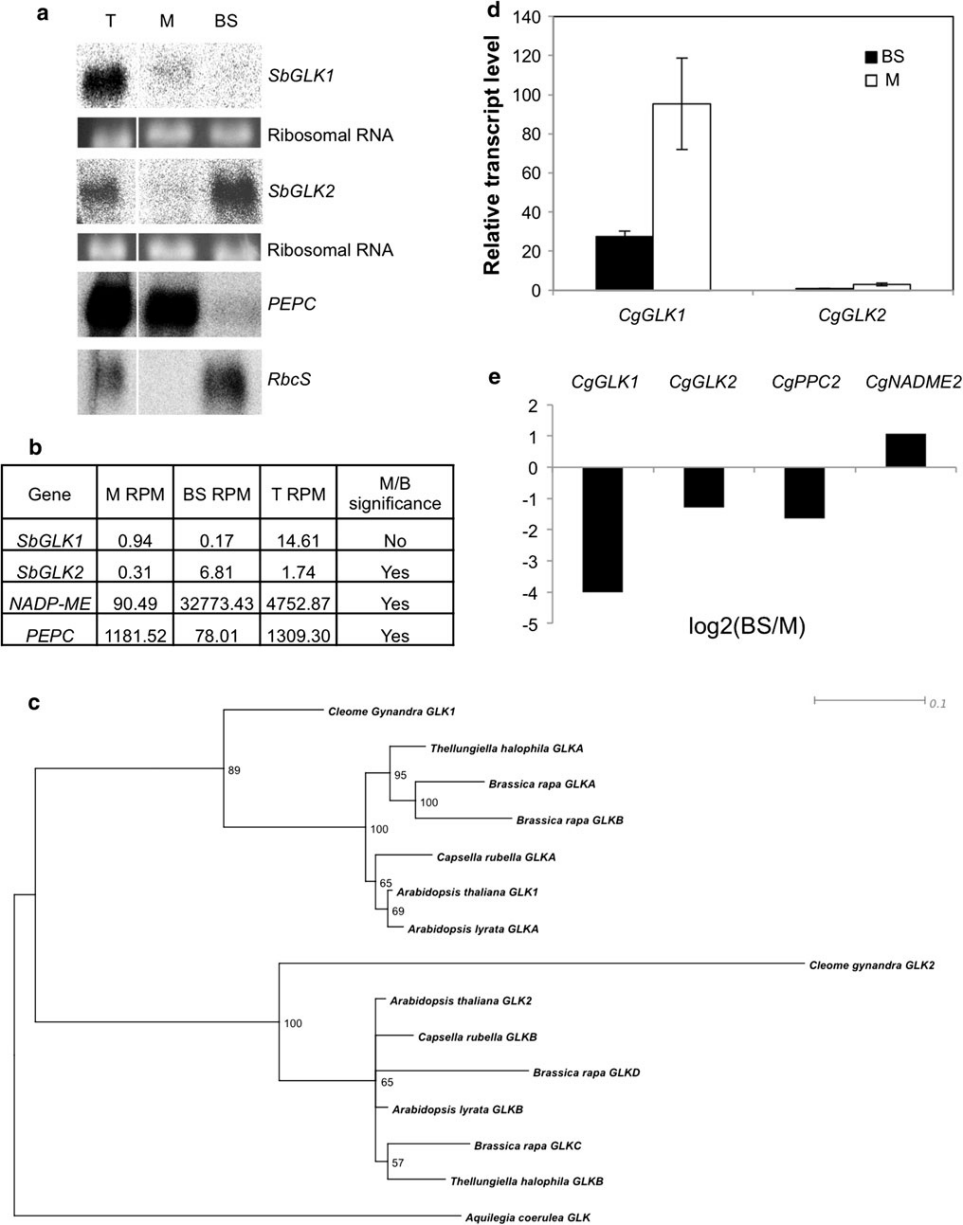
Analysis of *GLK* gene expression in leaves of *Sorghum bicolor* and *Cleome gynandra*. **a** Gel blot of 10 µg total RNA extracted from total leaf (T), mesophyll (M) or bundle sheath (BS) cells of sorghum. The blot was hybridized with *SbGLK1* and *SbGLK2,* and with maize *PEPC* (M cell-specific) and *RbcS* (BS cell-specific) sequences to confirm the purity of the cell preparations. Ethidium bromide stained ribosomal RNA bands are shown as loading controls. **b** Transcript levels of *SbGLK1* and *SbGLK2* in BS and M cells of sorghum as determined by 40 bp Illumina RNA sequencing, and quantified as reads per million (RPM) to two decimal places. Sorghum *PEPC* (M cell-specific) and *NADP*-*ME* (BS cell-specific) transcript levels demonstrate the purity of the cell extracts. Significance of differential gene expression between M and BS samples was calculated as described in the “Results”. **c** Bootstrapped maximum likelihood phylogenetic tree of a subset of *GLK* genes from the Brassicales with the Aquilegia (Ranunculales) gene used as the outgroup. **d** qPCR of *CgGLK1* and *CgGLK2* with RNA extracted from *C. gynandra* M and BS cells separated by LCM. Values are shown relative to *Actin7* transcript levels. *Bars* and *error bars* represent means and standard errors of three biological replicates, respectively. **e** Log2 of the ratio of BS/M transcript levels as determined by qPCR as in **c**. *CgPPC2* (M cell-specific) and *CgNADME2* (BS cell-specific) ratios confirm the purity of the cell preparations

To determine whether the cell-specific accumulation of *GLK* transcripts is a general feature of C_4_ biology rather than specific to maize, we carried out RNA gel blot and transcriptome analyses of sorghum BS and M cells. Figure 2a shows a blot analysis of RNA extracted from the two cell-types. As in maize, transcript levels of the sorghum ortholog of *ZmGlk1* (*SbGLK1*) are higher in M cells than BS cells, while transcripts of the sorghum ortholog of *ZmG2* (*SbGLK2*) accumulate preferentially in BS cells. Figure 2b shows similar results from Illumina sequencing of RNA extracted from sorghum M and BS cells. Using a significance test of differential gene expression (Audic and Claverie 1997) followed by a Bonferroni correction, *SbGLK2* transcript levels are shown to be significantly higher in BS than M cells. Although *SbGLK1* transcript levels are higher in M cells than BS cells, the difference is not significant by this test. However, this is a likely consequence of RNA turnover during the enzymatic digestion process for M cell separation, as suggested by comparing transcript levels in M cells with those in untreated total sorghum leaves (where both *SbGLK1* and *PEPC* are present at lower levels in M cells rather than being enriched as expected). Taken together, these data suggest that as in maize, *GLK* gene transcripts accumulate cell-specifically in sorghum.

Maize and sorghum share a common evolutionary origin of C_4_ photosynthesis (Christin et al. 2007). To determine whether there is similar cell-specific compartmentalization of *GLK* transcript accumulation in species with an independent origin of C_4_ photosynthesis and a separate trajectory of *GLK* duplication, we carried out qPCR on RNA isolated from BS and M cells of the C_4_ species *Cleome gynandra*. The eudicot *C. gynandra* is the closest C_4_ relative to *Arabidopsis* and it has two *GLK* genes that are orthologs of *AtGLK1* and *AtGLK2* (Fig. 2c). Transcripts of *CgGLK1* and *CgGLK2* can be detected in both BS and M cells, but levels of both are significantly higher in M cells (Fig. 2d, e). In both cell types, *CgGLK1* transcripts accumulate to tenfold higher level than *CgGLK2*. These observations suggest that compartmentalization of GLK function is not required for C_4_ chloroplast development in *C. gynandra.*

### Generation of *glk* mutants in rice

The *GLK* gene duplication in the Poales (asterisk in Fig. 1) preceded the speciation of rice, maize, and sorghum. In both maize and sorghum, transcript accumulation is compartmentalized and in maize this compartmentalization reflects cell-specific function. To determine whether the rice gene duplication also reflects sub-functionalization, single and double-mutant lines were generated.

**Fig. 3:**
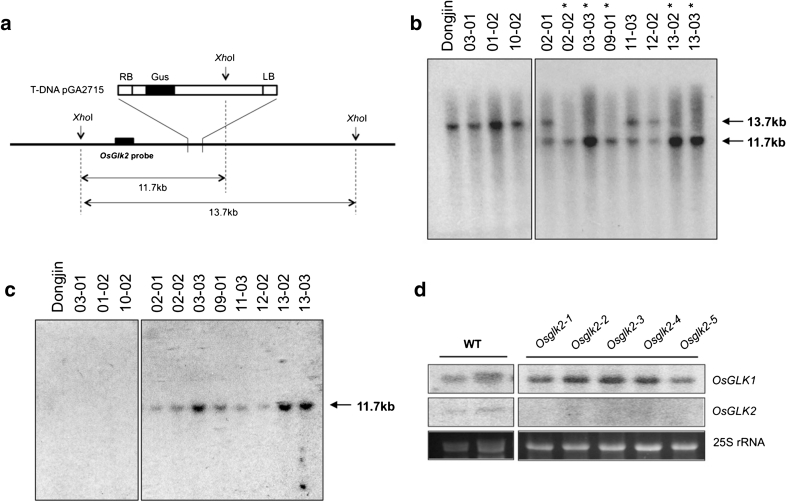
Identification of *Osglk2* single mutants. **a** Schematic of T-DNA insertion in the *OsGLK2* gene. *XhoI* restriction sites are indicated with *arrows*. Left and right T-DNA borders (LB, RB), the position of the B-glucuronidase (GUS) gene and of the *OsGLK2* fragment used for hybridization in **b** are shown. **b** Gel blot analysis of XhoI digested DNA from wild-type (Dongjin) and from lines segregating a T-DNA insertion in the *OsGLK2* gene. The position of the *OsGLK2* fragment used for hybridization is shown in **a**. *Asterisks* indicate homozygous mutant lines. **c** The same blot as in **b** hybridized with a GUS gene fragment to determine transgene copy number. **d** RNA gel blot analysis of replicate wild-type (WT), and *Osglk2* T_2_ single mutant lines. Blots were hybridized with both *OsGLK1* and *OsGLK2*. Ethidium bromide staining of 25S rRNA is shown as a loading control

An *Osglk2* insertion line was identified in a T-DNA tagged population (An et al. 2003). Fifteen segregating T_2_ lines (01–15) were first screened by PCR for the presence of the T-DNA (see “Materials and methods”). DNA extracted from 11 individuals representing eight of those lines was then hybridized to an *OsGLK2* gene fragment (Fig. 3b). Three individuals carried just the 13.7-kb fragment predicted for the wild-type Dongjin allele, five carried just the 11.7-kb fragment predicted for the insertion allele, and three carried both fragments. Further hybridization with a GUS gene fragment from the T-DNA insertion vector confirmed a single copy insertion of the T-DNA in the eight individuals containing the transgene (Fig. 3c). Five homozygous lines (02-02, 03-03, 09-01, 13-02, 13-03) that contain a single T-DNA insertion in the rice *OsGLK2* gene (Os01g13740) were therefore identified. We named these lines *Osglk2*-*1* to *Osglk2*-*5*, respectively. In all five lines, *OsGLK2* transcript levels were barely detectable by RNA gel blot analysis, whereas *OsGLK1* transcript levels were comparable to wild type (Fig. 3d).

**Fig. 4:**
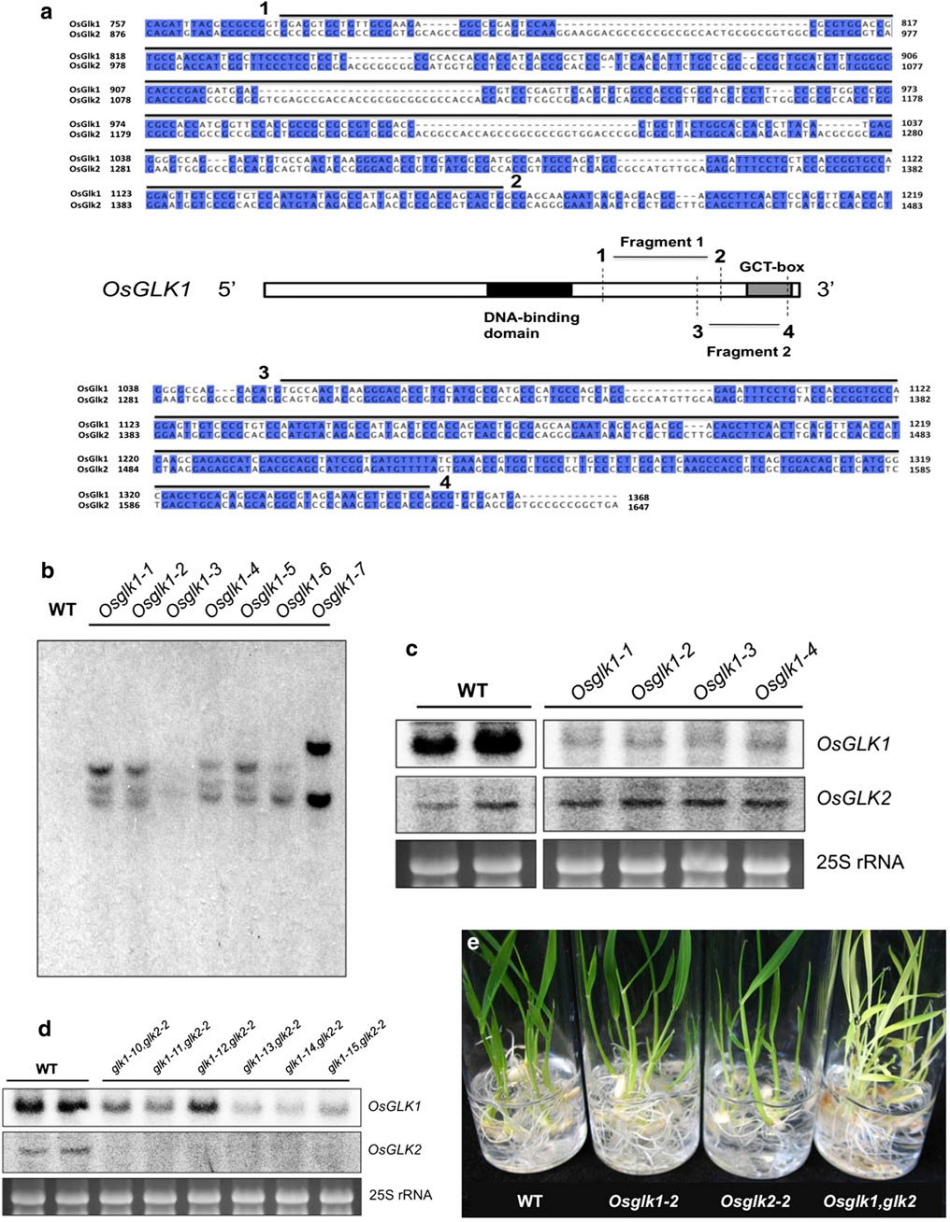
Generation of *Osglk1* and double-mutant lines. **a** Alignment of *OsGLK1* and *OsGLK2* sequences showing the position of the two fragments used for RNAi knockdown of *OsGLK1*. Fragment 1 is a 395 bp sequence between the DNA-binding domain and GCT-box, and fragment 2 is a 305 bp sequence spanning the GCT-box. **b** Gel blot of *Hin*dIII digested DNA from wild-type (WT) and T_1_ *Osglk1* knockdown lines. Blots were hybridized with an NPTII fragment from the transformation vector so that the number of hybridizing fragments would reveal the transgene copy number in the genome. **c** RNA gel blot analysis of replicate wild-type (WT) and *Osglk1* T_1_ single mutant lines. Blots were hybridized with both *OsGLK1* and *OsGLK2*. Ethidium bromide staining of 25S rRNA is shown as a loading control. **d** RNA gel blot analysis of replicate wild-type (WT) and T_0_ *Osglk1,glk2*-*2* double-mutant lines. Blots were hybridized with both *OsGLK1* and *OsGLK2*. Ethidium bromide staining of 25S rRNA is shown as a loading control. **e** Phenotype of 2 week old regenerated *Osglk1,glk2*-*2* double-mutant seedlings alongside wild-type (WT), *Osglk1*-*2* and *Osglk2*-*2* single mutant seedlings germinated from seeds

To generate an *Osglk1* single mutant in rice, an RNAi construct was generated to specifically target *OsGLK1*. Figure 4a demonstrates the sequence overlap between the gene-specific RNAi (fragment 2), *OsGLK1* and *OsGLK2* genes. Following transformation of wild-type callus, seven independent lines were generated. DNA gel blot analysis of these lines demonstrated that transgene copy number ranged from one to three (Fig. 4b) and RNA gel blot analysis of four of the lines revealed substantially lower *OsGLK1* transcript levels than in wild-type (Fig. 4c). *OsGLK2* transcript levels were comparable to wild-type in all four lines (Fig. 4c).

Double-mutant lines were generated by introducing an RNAi construct (containing fragment 1 in Fig. 4a) into callus of the *Osglk2*-*2* single mutant line. RNA gel blot analysis of six T_0_ double-mutant lines demonstrated the absence of *OsGLK2* transcripts and reduced levels of *OsGLK1* transcripts (Fig. 4d). The degree to which *OsGLK1* transcript levels were reduced varied between lines, presumably as a consequence of transgene copy number and/or position of transgene insertion. Unlike single mutants, the regenerated *Osglk1,glk2* double mutants were phenotypically pale (Fig. 4e). However, further characterization of the phenotype was hampered by the fact that the regenerated T_0_ plants failed to produce seed.

### Characterization of *Osglk1*-*2,glk2*-*2* double mutants

A segregating population of double-mutant plants was generated by crossing hemizygous *Osglk1*-*2* RNAi lines with homozygous *Osglk2*-*2* single mutant lines, and selfing the F_1_ progeny of the cross. A double-mutant plant in the segregating F_2_ population was subsequently selfed. The resultant F_3_ lines contained only double-mutant plants and thus the F_2_ parent was homozygous for both the *Osglk1*-*2* RNAi transgene and the *Osglk2*-*1* insertion allele.

**Fig. 5:**
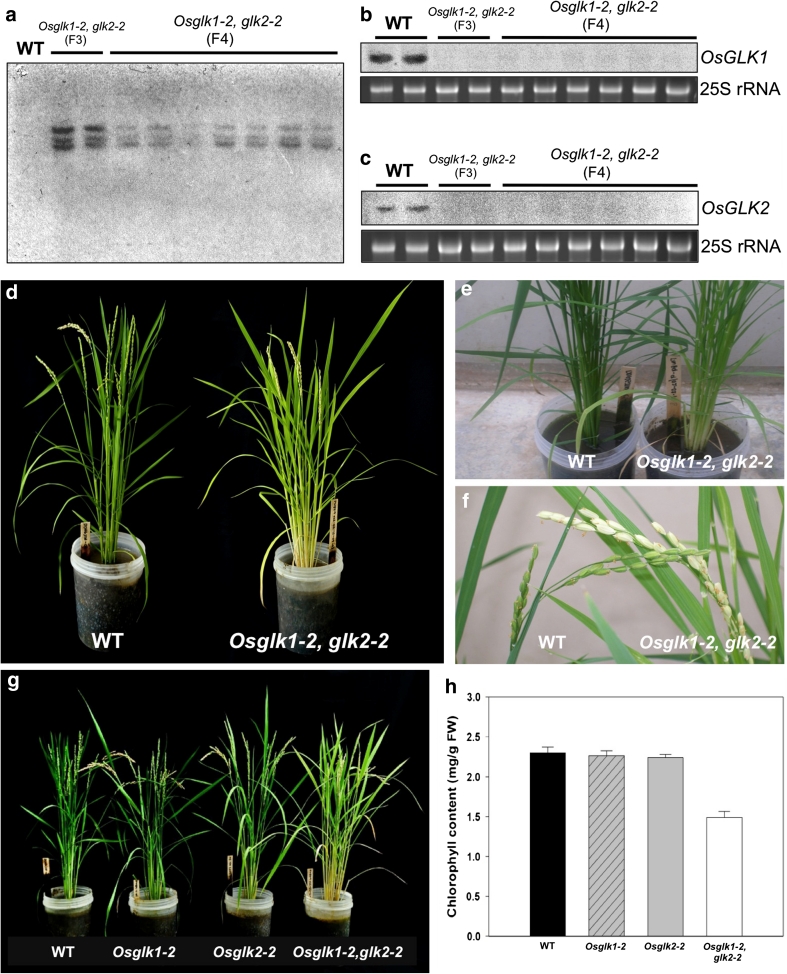
Characterization of *Osglk1*-*2, glk2*-*2* double mutants. **a** Gel blot analysis of *Hin*dIII digested DNA from wild-type plants and from F3 and F4 double-mutant plants. The blot was hybridized with an NPTII fragment to determine *OsGLK1* RNAi transgene copy number. **b**, **c** RNA gel blot analysis of replicate wild-type and double-mutant plants. Blots were hybridized with *OsGLK1* (**b**) and *OsGLK2* (**c**) fragments. Ethidium bromide staining of 25S rRNA is shown as a loading control. **d**–**f** Phenotype of wild-type and double-mutant plants demonstrating pale green leaves (**d**, **e**) and pale green inflorescences (**f**) in the double mutant. **g**, **h** Representative phenotypes of single and double mutants (**g**) used for measurement of chlorophyll content (**h**). Values are the average ± SE of 4 measurements. *FW* fresh weight

Given that the *Osglk1*-*2* RNAi line carries three copies of the *OsGLK1* RNAi transgene (Fig. 4b), DNA gel blot analysis was carried out to determine transgene copy number in F_3_ and F_4_ double-mutant lines. Figure 5a demonstrates that all nine double mutants examined carried three copies of the *OsGLK1* RNAi transgene. This observation suggests that the transgenes may be linked as they did not segregate in the F_1_ cross. RNA gel blot analysis of the same nine plants demonstrated that both *OsGLK1* (Fig. 5b) and *OsGLK2* (Fig. 5c) transcripts accumulate to reduced levels in double-mutant plants as compared with wild type. The extent to which transcript levels are reduced is comparable to that seen in regenerated double-mutant plants (compare *OsGLK1* hybridization signals in relation to amount of RNA loaded/WT hybridization signal in Figs. 4d, 5b, c). Unlike wild-type and single mutant plants, mature double mutants exhibit pale green leaf sheaths, leaf blades, and panicles (Fig. 5d–f). The relatively lower chlorophyll levels observed in double mutants by visual comparison of whole plants and leaf sections was confirmed by direct measurement. Figure 5h shows that chlorophyll levels are identical in wild-type and single mutants and that levels are ~65 % of wild-type in double mutants.

**Fig. 6:**
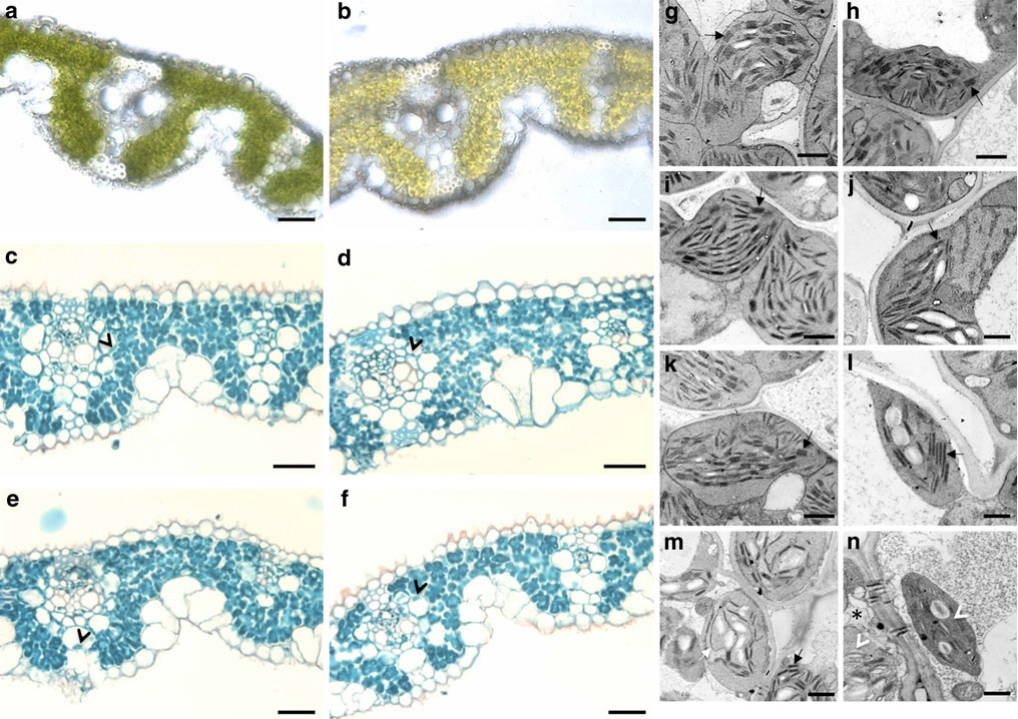
Leaf anatomy of *Osglk* single and double mutants. **a**–**f** Fresh (**a**, **b**) and safranin/fast green stained wax embedded (**c**–**f**) sections of wild-type (**a**, **c**), regenerated *Osglk1,glk2* double (**b**) *Osglk1*-*2,glk2*-*2* double (**d**), *Osglk1*-*2* single (**e**) and *Osglk2*-*2* single (**f**) mutants. *Black arrowheads* point to BS cell chloroplasts. *Scale bar* 50 µm. **g**–**n** Transmission electron micrographs of chloroplasts in M cells (**g**, **i**, **k**, **m**) and BS cells (**h**, **j**, **l**, **n**) of wild-type (**g**, **h**), *Osglk1*-*2* single mutant (**i**, **j**), *Osglk2*-*2* single mutant (**k**, **l**) and *Osglk1*-*2,glk2*-*2* double mutant (**m**, **n**). *Asterisks* in **n** denotes an adjoining M cell. *Black arrows* point to granal lamellae; *white arrow* to disorganized lamellae and *white arrowheads* to vesicles. *Scale bar* 1 µm

To determine the extent to which chloroplast development is perturbed in single and double-mutant plants, leaf anatomy was examined by both light and transmission electron microscopy (TEM). In thick leaf sections, reduced chlorophyll levels are apparent in double mutants (Fig. 6a, b), and in thin sections reduced chloroplast size is observed in both BS and M cells of double *Osglk1*-*2,glk2*-*2* mutants (Fig. 6c, d) but not in the *Osglk1*-*2* RNAi line (Fig. 6e) or in the *Osglk2*-*2* single mutant (Fig. 6f). The smaller chloroplast size in double mutants was confirmed by TEM (representative images in Fig. 6g–n). TEMs further demonstrated that in wild-type (Fig. 6g, h) and single mutants (Fig. 6i–l) both M and BS chloroplasts exhibit granal lamellae. The size of individual granal stacks is roughly equivalent in the two chloroplast types but given that M chloroplasts are generally larger than BS chloroplasts, the overall granal volume is greater in M cells. In double mutants, some chloroplasts appear relatively normal (e.g. Fig. 6m, lower right) but in most cases only rudimentary thylakoids develop (Fig. 6m, n). This perturbation to membrane topology is accompanied by the accumulation of vesicles within both M and BS chloroplasts (Fig. 6m, n). Therefore, despite being orthologs of the cell-specific *GLK* genes in maize and sorghum, *OsGLK1* and *OsGLK2* regulate chloroplast development in both BS and M cells.

## Discussion

As land plants evolved from aquatic green algae, the GARP superfamily of transcription factors expanded through multiple gene duplications. This is evidenced by the fact that the sequenced genomes of the extant green algae *Chlamydomonas reinhardtii* and *Volvox carteri* contain four GARP genes, whereas those of the flowering plants *Arabidopsis* and maize contain 54 and 98 respectively (Riechmann et al. 2000; Plant Transcription Factor Database http://planttfdb.cbi.edu.cn/family.php?fam=G2-like). In land plants, the *GLK* gene members of the GARP family vary in copy number from one to four (Fig. 1) but no *GLK* genes are present in sequenced algal genomes. It is thus likely that *GLK* genes evolved through modification of GARP sequences prior to, or concomitantly with, the transition to land.

Based on current evidence, it is most likely that ancestral land plants had a single *GLK* gene. Preliminary data suggest that this ancestral state is retained in the genomes of the extant hornwort *Anthoceros punctatus* (E. Frangedakis, S. Kelly, J. Fouracre and JA Langdale, unpublished data) and the extant liverwort *Marchantia polymorpha* (Kimitsune Ishizaki, Kyoto University, Plant Mol Biol Lab, Kyoto, Japan, personal communication). Although two genes are present in the moss *P. patens,* phylogenetic analyses indicate that these are the result of a recent genome duplication within that species rather than a gene-specific duplication (Yasumura et al. 2005; Rensing et al. 2008). The proposed ancestral single gene state is also retained in the lycophyte *S. moellendorffii*. Unfortunately, the paucity of genome sequence in other non-seed plants precludes further speculation on the timing of *GLK* gene duplication events prior to the divergence of the angiosperms.

Within the angiosperms, the topology of the *GLK* gene tree reflects the multiple genome-wide duplications (GWD) that have occurred in the group (reviewed in Soltis et al. 2009). In the eudicots, patterns of gene duplication are complex but can be rationalized as follows. First, all of the observed *GLK* gene duplications post-date the ancient hexaploidization event that occurred before the divergence of the Rosids and Asterids (Jaillion et al. 2007) because orthologous *GLK* gene relationships cannot be demonstrated between species of the two groups. In the Rosales, the two *GLK* genes in *M. domestica* reflect a family specific GWD within the Maleae tribe (Velasco et al. 2010). In the Fabiales, two GWD events within the legumes—one around 54 million years ago before the divergence of soybean and common bean from Medicago and one around 13 million years ago within soybean (Cannon et al. 2010; Schmutz et al. 2010)—explain the presence of two *GLK* genes in the genome of *P. vulgaris* and four genes in the *G. max* genome. The single gene in *M. trunculata* infers gene loss in that species sometime after the original legume duplication. In the Malpighiales, the two *GLK* genes in *P. trichocarpa* reflect a family specific GWD within the Salicaceae (Tuskan et al. 2006) and the three *GLK* genes in *L. usitatissimum* suggest within-species duplications. The two *GLK* genes in *M. esculenta* and the single gene in *R. communis* support a duplication within the Euphorbiaceae followed by gene loss in *R. communis*.

The specific evolutionary trajectories leading to duplicate *GLK* genes in the C_4_ eudicot *C. gynandra* and the C_4_ monocots maize and sorghum, can be rationalized as follows. In the Brassicales, there is one *GLK* gene in *C. papaya*, two genes in four of the other sequenced genomes and four genes in the *Brassica rapa* genome. The topology of the gene tree in Fig. 1 suggests that the original duplication resulted from the GWD that occurred after the divergence of Capparaceae from Brassicaceae and Cleomaceae, but prior to the divergence of *Arabidopsis* and *B. rapa* (Blanc et al. 2003), and that a subsequent GWD occurred within *B. rapa*. Despite reports of independent GWD in the Cleomaceae and Brassicaceae (Schranz and Mitchell-Olds 2006), our phylogenetic evidence indicates that the *C. gynandra* *GLK* genes are orthologs of the *Arabidopsis* genes (Fig. 2c). Thus, *GLK* gene duplication occurred prior to the evolution of C_4_ within the Brassicales. In the monocots the situation is similar but more straightforward. The six sequenced monocot genomes represent genera in the order Poales. Given that all six species contain two *GLK* genes, and that the tree robustly resolves orthologous and paralogous relationships (Fig. 1), it is clear that a single duplication occurred prior to speciation in this group and hence prior to the evolution of C_4_. This observation is consistent with the reported GWD in the Poales (reviewed in Soltis et al. 2009). Given that the single *GLK* genes in the genomes of *C. sativus*, *A. coerulea*, *P. persica*, *C. sinensis* and *V. vinifera* correlate with the absence of C_4_ species in the respective orders (Cucurbitales, Ranunculales, Rosales, Sapindales, Vitales) (Sage et al. 2011), it is tempting to speculate that *GLK* gene duplication was a prerequisite for C_4_ evolution. Notably, although a single gene is present in *R. communis,* and C_4_ species are present in the Euphorbiaceae, gene loss is inferred in this case as discussed above. More genome sampling is required to confirm or refute the suggestion that *GLK* gene duplication preconditions C_4_, and to address the importance of gene duplication for the evolution of C_4_ photosynthesis in general (Monson 2003; Williams et al. 2012).

The presence of two *GLK* genes in maize and sorghum is associated with compartmentalization of *GLK* gene activity in BS and M cells, suggesting that each gene may have a cell-type specific function in C_4_ plants more generally (Rossini et al. 2001). In the C_3_ plant *Arabidopsis*, GLK transcription factors act cell-autonomously to regulate a suite of genes involved in light harvesting and chlorophyll biosynthesis (Waters et al. 2008, 2009). In so doing, GLK activity modulates thylakoid stacking and the assembly of photosystem complexes. In both maize and sorghum, BS and M cell chloroplasts exhibit different degrees of thylakoid stacking and different compositions of photosystems. PSI functions in agranal BS chloroplasts whereas both PSI and PSII function in granal M chloroplasts. These differences could result from specialized cell autonomous activities of the compartmentalized GLK proteins or could be mediated through interactions between GLK proteins and BS or M cell-specific partner proteins. The latter suggestion is certainly plausible given that the two *Arabidopsis* GLK proteins have been shown to hetero- and homo-dimerize (Rossini et al. 2001) and to interact with G-box binding proteins (Tamai et al. 2002).

Whilst the cell-specific role of *GLK* genes in maize and sorghum is consistent with the suggestion that compartmentalization of the two proteins is required for chloroplast development in C_4_ plants, cell-specific accumulation of *GLK* gene transcripts was not detected in BS and M cells of the C_4_ eudicot *C. gynandra* (Fig. 2d, e). It is possible that cell-specific activity of GLK proteins is regulated post-transcriptionally in *C. gynandra*. However, given that both BS and M chloroplasts of *C*. *gynandra* are granal (Marshall et al. 2007), and hence less morphologically distinct than those of maize and sorghum, it is also possible that there is no need for specialization in this species. Compartmentalized GLK function may thus be restricted to C_4_ species with dimorphic chloroplasts. Such dimorphism is found in chloroplasts of both C_4_ eudicots and monocots (Laetsch 1974).

In most species examined, genomes containing more than one *GLK* gene have undergone a recent GWD event. Given that such events are normally followed by progressive diploidization and the reduction of DNA content (Wolfe 2001), the question remains as to why *GLK* gene pairs persist in C_3_ species where they essentially function redundantly to regulate chloroplast development in all photosynthetic cells of the leaf (Figs. 4, 5, 6; Fitter et al. 2002; Yasumura et al. 2005). Because the proposed role of *GLK* genes is to balance the light and dark reactions of photosynthesis in order to optimize carbon fixation (reviewed in Waters and Langdale 2009), we hypothesize that in C_3_ species with multiple *GLK* genes, some degree of sub-functionalization has occurred. This suggestion is supported by recent studies demonstrating differential responses of the two *GLK* genes in *Arabidopsis* to organic nitrogen (Gutiérrez et al. 2008), perturbed plastid import pathways (Kakizaki et al. 2009) and cytokinin (Kobayashi et al. 2012). Some developmental specialization can also be seen in that only *At*GLK2 functions in the siliques of *Arabidopsis* (Fitter et al. 2002). These observations therefore suggest that in both C_3_ and C_4_ plants, the coordinated and combined activity of GLK proteins acts to integrate environmental and developmental signals to maximize carbon assimilation.

## Electronic supplementary material

Below is the link to the electronic supplementary material.

Supplementary material 1 (XLSX 54 kb)

## Abbreviations

BS: Bundle sheath
GLK: GOLDEN2-LIKE
M: Mesophyll

## References

[CR1] An S, Park S, Jeong DH (2003). Generation and analysis of end sequence database for T-DNA tagging lines in rice. Plant Physiol.

[CR2] Arnon DI (1949). Copper enzymes in isolated chloroplast polyphenoloxidase in *Beta vulgaris*. Plant Physiol.

[CR3] Audic S, Claverie JM (1997). The significance of digital gene expression profiles. Genome Res.

[CR4] Blanc G, Hokamp K, Wolfe KH (2003). A recent polyploidy superimposed on older large-scale duplications in the *Arabidopsis* genome. Genome Res.

[CR5] Bravo-Garcia A, Yasumura Y, Langdale JA (2009). Specialization of the *Golden2*-*like* regulatory pathway during land plant evolution. New Phytol.

[CR6] Cannon SB, Ilut D, Farmer AD, Maki SL, May GD, Singer SR, Doyle JJ (2010). Polyploidy did not predate the evolution of nodulation in all legumes. PLoS One.

[CR7] Christin PA, Salamin N, Savolainen V, Duvall MR, Besnard G (2007). C_4_ photosynthesis evolved in grasses via parallel adaptive genetic changes. Curr Biol.

[CR8] Collingridge PW, Kelly S (2012). MergeAlign: improving multiple sequence alignment performance by dynamic reconstruction of consensus multiple sequence alignments. BMC Bioinforma.

[CR9] Eddy SR (1998). Profile hidden Markov models. Bioinformatics.

[CR10] Fang J, Chai C, Qian Q, Li C, Tang J, Sun L, Huang Z, Guo X, Sun C, Liu M, Zhang Y, Lu Q, Wang Y, Lu C, Han B, Chen F, Cheng Z, Chu C (2008). Mutations of genes in synthesis of the carotenoid precursors of ABA lead to pre-harvest sprouting and photo-oxidation in rice. Plant J.

[CR11] Fitter DW, Martin DJ, Copley MJ, Scotland RW, Langdale JA (2002). *GLK* gene pairs regulate chloroplast development in diverse plant species. Plant J.

[CR12] Galtier N (2001). Maximum-likelihood phylogenetic analysis under a covarion-like model. Mol Biol Evol.

[CR13] Gutiérrez RA, Stokes TL, Thum K, Xu X, Obertello M, Katari MS, Tanurdzic M, Dean A, Nero DC, McClung CR, Coruzzi GM (2008). Systems approach identifies an organic nitrogen-responsive gene network that is regulated by the master clock control gene *CCA1*. Proc Natl Acad Sci USA.

[CR14] Hall LN, Rossini L, Cribb L, Langdale JA (1998). GOLDEN2: a novel transcriptional regulator of cellular differentiation in the maize leaf. Plant Cell.

[CR15] Huelsenbeck JP, Ronquist F (2001). MRBAYES: Bayesian inference of phylogenetic trees. Bioinformatics.

[CR16] Jaillion O, Aury J-M, Noel B (2007). The grapevine genome sequence suggests ancestral hexaploidization in major angiosperm phyla. Nature.

[CR17] Kakizaki T, Matsumura H, Nakayama K, Che FS, Terauchi R, Inaba T (2009). Coordination of plastid protein import and nuclear gene expression by plastid-to-nucleus retrograde signaling. Plant Physiol.

[CR18] Katoh K, Kuma K, Miyata T, Toh H (2005). Improvement in the accuracy of multiple sequence alignment program MAFFT. Genome Inform.

[CR19] Kelly S, Wickstead B, Gull K (2011). Archaeal phylogenomics provides evidence in support of a methanogenic origin of the Archaea and a thaumarchaeal origin for the eukaryotes. Proc Biol Sci.

[CR20] Kobayashi K, Baba S, Obayashi T, Sato M, Toyooka K, Keranen M, Aro EM, Fukaki H, Ohta H, Sugimoto K, Masuda T (2012). Regulation of root greening by light and auxin/cytokinin signaling in *Arabidopsis*. Plant Cell.

[CR21] Laetsch WM (1974). The C_4_ syndrome: a structural analysis. Annu Rev Plant Physiol.

[CR22] Langdale JA, Freeling M, Walbot V (1994). In situ hybridization. The maize handbook.

[CR23] Langdale JA (2011). C_4_ cycles: past, present, and future research on C_4_ photosynthesis. Plant Cell.

[CR24] Langdale JA, Kidner CA (1994). *bundle sheath defective*, a mutation that disrupts cellular differentiation in maize leaves. Development.

[CR25] Langdale JA, Rothermel BA, Nelson T (1988). Cellular patterns of photosynthetic gene expression in developing maize leaves. Genes Dev.

[CR26] Langdale JA, Zelitch I, Miller E, Nelson T (1988). Cell position and light influence C_4_ versus C_3_ patterns of photosynthetic gene expression in maize. EMBO J.

[CR27] Langmead B, Trapnell C, Pop M, Salzberg SL (2009). Ultrafast and memory-efficient alignment of short DNA sequences to the human genome. Genome Biol.

[CR28] Le SQ, Gascuel O (2008). An improved general amino acid replacement matrix. Mol Biol Evol.

[CR29] Marshall DM, Muhaidat R, Brown NJ, Liu Z, Stanley S, Griffiths H, Sage RF, Hibberd JM (2007). *Cleome*, a genus closely related to *Arabidopsis*, contains species spanning a developmental progression from C_3_ to C_4_ photosynthesis. Plant J.

[CR30] Monson RK (2003). Gene duplication, neofunctionalization, and the evolution of C_4_ photosynthesis. Int J Plant Sci.

[CR31] Murray MG, Thompson WF (1980). Rapid isolation of high molecular weight plant DNA. Nucleic Acids Res.

[CR32] Nakamura H, Muramatsu M, Hakata M, Ueno O, Nagamura Y, Hirochika H, Takano M, Ichikawa H (2009). Ectopic overexpression of the transcription factor OsGLK1 induces chloroplast development in non-green rice cells. Plant Cell Physiol.

[CR33] Nishimura A, Aichi I, Matsuoka M (2006). A protocol for *Agrobacterium*-mediated transformation in rice. Nat Protoc.

[CR34] Rensing SA, Lang D, Zimmer AD (2008). The *Physcomitrella* genome reveals evolutionary insights into the conquest of land by plants. Science.

[CR35] Riechmann J, Heard J, Martin G, Reuber L, Jiang C, Keddie J, Adam L, Pineda O, Ratcliffe O, Samaha R, Creelman R, Pilgrim M, Broun P, Zhang J, Ghandehari D, Sherman B, Yu G (2000). *Arabidopsis* transcription factors: genome-wide comparative analysis among eukaryotes. Science.

[CR36] Rossini L, Cribb L, Martin DJ, Langdale JA (2001). The maize *golden2* gene defines a novel class of transcriptional regulators in plants. Plant Cell.

[CR37] Sage RF, Christin P-A, Edwards EJ (2011). The C_4_ plant lineages of planet earth. J Exp Bot.

[CR38] Schmutz J, Cannon SB, Schlueter J (2010). Genome sequence of the palaeopolyploid soybean. Nature.

[CR39] Schranz ME, Mitchell-Olds T (2006). Independent ancient polyploidy events in the sister families Brassicaceae and Cleomaceae. Plant Cell.

[CR40] Sheen JY, Bogorad L (1985). Differential expression of the ribulose bisphosphate carboxylase large subunit gene in bundle sheath and mesophyll cells of developing maize leaves is influenced by light. Plant Physiol.

[CR41] Soltis DE, Albert VA, Leebens-Mack J, Bell CD, Paterson AH, Zheng C, Sankoff D, de Pamphilis CW, Wall PK, Soltis PS (2009). Polyploidy and angiosperm diversification. Am J Bot.

[CR42] Stamatakis A (2006). RAxML-VI-HPC: maximum likelihood-based phylogenetic analyses with thousands of taxa and mixed models. Bioinformatics.

[CR43] Sukumaran J, Holder MT (2010). DendroPy: a Python library for phylogenetic computing. Bioinformatics.

[CR44] Tamai H, Iwabuchi M, Meshi T (2002). *Arabidopsis* GARP transcriptional activators interact with the Pro-rich activation domain shared by G-Box-binding bZIP factors. Plant Cell Physiol.

[CR45] Tuskan GA, Difazio S, Jansson S (2006). The genome of black cottonwood, *Populus trichocarpa* (Torr. & Gray). Science.

[CR46] Velasco R, Zharkikh A, Affourtit J (2010). The genome of the domesticated apple (*Malus x domestica* Borkh.). Nat Genet.

[CR47] Waters MT, Langdale JA (2009). The making of a chloroplast. EMBO J.

[CR48] Waters MT, Moylan EC, Langdale JA (2008). GLK transcription factors regulate chloroplast development in a cell-autonomous manner. Plant J.

[CR49] Waters MT, Wang P, Korkaric M, Capper RG, Saunders NJ, Langdale JA (2009). GLK transcription factors coordinate expression of the photosynthetic apparatus in *Arabidopsis*. Plant Cell.

[CR50] Westhoff P, Offermannsteinhard K, Hofer M, Eskins K, Oswald A, Streubel M (1991). Differential accumulation of plastid transcripts encoding photosystem-II components in the mesophyll and bundle-sheath cells of monocotyledonous NADP-malic enzyme-type-C_4_ plants. Planta.

[CR51] Williams BP, Aubry S, Hibberd JM (2012). Molecular evolution of genes recruited into C_4_ photosynthesis. Trends Plant Sci.

[CR52] Wolfe KH (2001). Yesterday’s polyploids and the mystery of diploidization. Nat Rev Genet.

[CR53] Wyrich R, Dressen U, Brockmann S, Streubel M, Chang C, Qiang D, Paterson A, Westhoff P (1998). The molecular basis of C_4_ photosynthesis in sorghum: isolation, characterization and RFLP mapping of mesophyll- and bundle-sheath-specific cDNAs obtained by differential screening. Plant Mol Biol.

[CR54] Yamada M, Kawasaki M, Sugiyama T, Miyake H, Taniguchi M (2009). Differential positioning of C_4_ mesophyll and bundle sheath chloroplasts: aggregative movement of C_4_ mesophyll chloroplasts in response to environmental stresses. Plant Cell Physiol.

[CR55] Yasumura Y, Moylan E, Langdale J (2005). A conserved transcription factor mediates nuclear control of organelle biogenesis in anciently diverged land plants. Plant Cell.

